# Synthesis of Hollow Spherical Phosphide Catalysts with Industrial-Scale Potential for Alkaline Hydrogen Evolution Reaction

**DOI:** 10.1021/acsami.5c11704

**Published:** 2025-09-22

**Authors:** Magdalena Streckova, Alena Fedorockova, Alexandra Guboova, Gabriel Sučik, Vladimir Girman, Akbar Hussain, Michael Vorochta, Jozef Strečka, Tomas Bystron

**Affiliations:** † Institute of Materials Research, Slovak Academy of Sciences, Watsonova 47, 040 01 Kosice, Slovak Republic; ‡ Faculty of Materials, Metallurgy and Recycling, Technical University of Kosice, Letna 9, 042 00 Kosice, Slovakia; § Institute of Physics, Faculty of Science, P.J. Safarik University, Park Angelinum 9, 041 01 Kosice, Slovak Republic; ∥ Department of Chemistry, 66757 Quaid-i-Azam University, 45320 Islamabad, Pakistan; ⊥ Department of Physical Chemistry, Faculty of Science, P.J. Šafárik University, Moyzesova 11, SK-04154 Košice, Slovak Republic; # Department of Surface and Plasma Science, Faculty of Mathematics and Physics, Charles University, V Holešovičkách 2, 18000 Prague 8, Czech Republic; ¶ Department of Theoretical Physics and Astrophysics, Faculty of Science, P.J. Šafárik University, Park Angelinum 9, 040 01 Košice, Slovak Republic; ∇ Department of Inorganic Technology, 52735 University of Chemistry and Technology Prague, Technicka 5, 166 28 Prague 6, Czech Republic

**Keywords:** hydrogen evolution reaction, electrocatalysis, water electrolysis, transition metal phosphides, activation energy

## Abstract

The competitiveness of hydrogen as a fuel of the future relies on the development of low-cost, readily available, and efficient catalysts. Transition metal phosphides (TMP), mainly in multimetallic compositions, are recognized as an excellent alternative to platinum-based catalysts in electrolyzers and fuel cells. Herein, for the first time, the connection of the spray drying method as a user-friendly technique for the large-scale production of TMP as hydrogen evolution reaction (HER) catalysts is presented. Three types of catalysts based on MoFeP modified with Co and Ni as third elements were compared by electrochemical methods in an alkaline medium of 1 M KOH. Among the studied materials, the as-prepared MoFeCoP electrocatalysts exhibited the lowest reaction overpotentials (η_10_) of −285 mV, with Tafel slopes of 83 mV dec^–1^, low activation energy for the HER and sufficient durability in long-term stability tests. The high value of differential capacitance for both trimetallic materials MoFeNiP and MoFeCoP indicates a large surface area, resulting in excellent features for hydrogen production and application.

## Graphical abstract

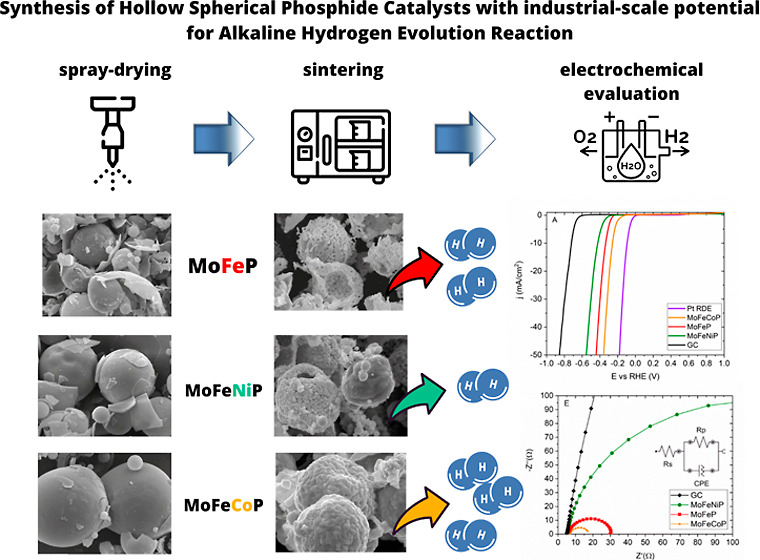

## 1 Introduction

The increase in global energy demand is driven by a number of socioeconomic factors, such as population growth, urbanization, industrial progress, the development of new technologies, and rising net capital income. This is expected to continue to grow rapidly, with demand potentially doubling by 2050. Global dependence on fossil fuels as a primary energy source poses significant environmental and health risks due to greenhouse gas emissions.^1^ As readily available fossil fuel reserves dwindle, their extraction is becoming increasingly difficult and expensive.

Hydrogen is an efficient energy carrier and storage medium, with numerous benefits. A key advantage is its suitability for long-term storage in practically unlimited amounts. Through electrolysis, excess renewable energy can be used to produce hydrogen gas, allowing for efficient and sustained storage with minimal energy loss. This provides a scalable solution to address the intermittent nature of renewable energy sources.^2^ In general, electrochemical water splitting (eq 1), as a nonspontaneous process, is driven by the application of electrical energy. It proceeds as two half-reactions: the hydrogen evolution reaction (HER) and oxygen evolution reaction (OER).

1 $$H_{2}O\to 2H_{2\left(g\right)}+1/2O_{2\left(g\right)}$$

Under alkaline conditions, hydroxide anions produced by reductive decomposition of water at the cathode (eq 2) are transported toward the anode, where they are oxidized to O_2_ (eq 3).

2 $$At\;the\;cathode\;\left(reduction\right):\;2H_{2}O+{2e}^{-}\to H_{2\left(g\right)}+{2OH}^{-}$$

3 $$At\;the\;anode\;\left(oxidation\right):\;2{OH}^{-}\to 1/2O_{2\left(g\right)}+H_{2}O+2e^{-}$$

Within electrolysis technologies, four common methods are currently known: alkaline water electrolysis (AWE), anion exchange membrane electrolysis, proton exchange membrane electrolysis, and solid oxide electrolysis. Among these, AWE remains the most advanced and historically the most reliable, cost-effective, and mature technology for large-scale hydrogen production.^3^ Despite the maturity, robustness, and worldwide deployment, it has severe limitations. The most important ones are the low flexibility of operation and inefficient operation at low current densities. Therefore, ongoing research is focused on addressing these issues. One of the investigated approaches, allowing for improvement of the process performance parameters, is the application of new electrode materials with superior catalytic activity and stability. This can be achieved through regulated composition, morphology, and nanostructure. Such catalysts could significantly reduce overpotential and enhance the long-term electrocatalytic stability and kinetics of overall water electrolysis.^4,5^

In recent years, transition metal phosphides (TMPs) have been confirmed as promising alternatives to costly precious metal-based electrocatalysts with strong electrocatalytic activity.^6^ The enhanced electrocatalytic activity of TMP for HER is attributed to the abundant catalytically active sites and the synergistic effect of phosphorus and metallic atoms, which optimize the electronic structure of the surface, providing suitable free adsorption energy of key intermediates.^6,7^ Additionally, TMP based on nickel (Ni) exhibit multielectron orbitals that attract protons and possess metalloid properties due to the delocalized electron cloud from the Ni–Ni metallic bonds. The presence of phosphorus atoms (P) introduces ionic bonds (Ni^δ+^–P^δ−^), which facilitate hydrogen release and enhance stability and resistance in both acidic and highly alkaline environments. Furthermore, Ni_*x*_P_*y*_ materials have the ability to expose more unsaturated surface atoms, offering increased catalytic potential.^7^

Yin et al. compared the electrochemical performance of five typical monometallic TMPs (Ni_*x*_P_*y*_, Co_*x*_P_*y*_, Mo_*x*_P_*y*_, W_*x*_P_*y*_, and Fe_*x*_P_*y*_) at different degrees of phosphorization and with different nanostructures.^8^ It is clear from the review that phosphorus content, crystallinity, and morphology have an important influence on HER activity. The comparison shows that Ni_*x*_P_*y*_ and Co_*x*_P_*y*_ exhibit good electrocatalytic performance. For example, Ni_12_P_5_ nanoneedles with lower phosphorus content achieve in 1.0 M KOH an overpotential of −59 mV at 10 mA cm^‑2^, while porous CoP nanoparticles show an overpotential of −35 mV for the same current density in 1.0 M KOH, but their stability is not as high as that of Mo_*x*_P_*y*_ and W_*x*_P_*y*_. Among the compared monometallic phosphides, Fe_*x*_P_*y*_ was characterized by the lowest cost and decent electrocatalytic activity, while the activity of Mo_*x*_P_*y*_ and W_*x*_P_*y*_ still requires further improvement. Recent investigations into TMP have shown significant advancements via heterostructuring strategies to improve the catalytic properties. For example, Wu et al. reviewed the use of phosphide heterostructures in oxygen evolution, synthesizing best practices for enhanced active-site exposure and interfacial engineering,^9^ and developed phase-controllable CoP–Co_2_P heterostructures showing impressive activity and stability for hydrogen production in both water and seawater, highlighting the importance of phase synergy and surface reconstruction.^10^

Based on this knowledge, it is clear that elemental doping forming a multimetallic phosphide structure enables the modulation of materials at the lattice level, enhancing both the electrocatalytic activity and stability. This approach will be a key direction of future development, as it involves employing new strategies to regulate the microstructure. The three most significant advantages of multimetallic TMPs in electrochemical performance for electrochemical water splitting were described in detail in the review of Zhang et al.^11^

- High electronic conductivity is crucial for electrode materials to achieve high power density and for electrocatalysts to enable fast reaction kinetics. Multimetallic TMPs meet this requirement, offering higher conductivity than their monometallic counterparts, as suggested by DFT calculations showing increased density of states near the Fermi level.^10–12^

- Introducing secondary metals alters the electronic structure of TMP via ligand and strain effects. The ligand effect redistributes valence electrons, creating more active sites for redox reactions, while the strain effect modifies adsorption energy and the d-band center, leading to a favorable electronic structure.^13–16^

- Introducing secondary metals into monometallic TMPs can create unique nanostructures, activating inert sites to enhance electrochemical performance. The most studied bimetallic catalysts are based on NiCoP,^17,18^ FeCoP,^19,20^ and NiFeP.^21^

Han et al. designed CoNiP nanospheres on a 3D Ni foam current collector as a self-standing cathode for an efficient HER across a wide pH range. Specifically, the overpotentials (η) at a current density of −10 mA cm^–2^ were −60, −120, and −155 mV under acidic, neutral, and alkaline conditions, respectively.^17^ Bera et al. reported the preparation of highly innovative material based on self-standing NiCoP fibers. Electrochemical tests revealed that the NiCoP fibers prepared by the needleless electrospinning technology possess decent electrocatalytic activity for HER in alkaline solution, reaching low overpotentials (η_–10_ = −141 mV and η_–20_ = −230 mV) and a low value of Tafel slope (66 mV dec^–1^). In acidic media, the corresponding values are η_–10_ = −146 mV, η-_20_ = −265 mV, and a Tafel slope ≈ 77 mV dec^–1^.^18^

As mentioned above, most active TMP-based HER catalysts often possess Co. However, the widespread use of Co is limited due to its high price, concerns about raw material availability, and ethical issues of using child labor during mining.^22,23^ Therefore, reducing the amount of Co with more accessible and cheaper 3d elements, such as Ni, is highly desirable. Improving the electrochemical properties of bimetallic phosphides by drawing insights from their monometallic counterparts has led to the incorporation of additional metals into the crystal lattice, forming trimetallic phosphides. For instance, a series of homogeneous trimetallic Fe_*x*_Co_*y*_Ni_*z*_P on carbon cloth were designed by Gu et al. for HER in an acidic electrolyte.^24^ They found that the equimolar metal ratio in Fe_0.33_Co_0.33_Ni_0.33_P leads to enhanced HER activity, as was predicted by DFT calculations. Qian et al. prepared nanoporous NiFeMoP by an electrospinning process followed by chemical etching. Due to the beneficial continuous porous structure and the synergetic effect between Mo and other metals, bifunctional electrocatalytic activity was observed. Particularly, η_20_ of only 193 mV and a Tafel slope of 41.2 mV dec^–1^ were achieved for the OER in 1 M KOH. The authors reported an extremely low cell voltage of 1.41 V for overall water splitting at a current density of 10 mA cm^–2^.^25^ Inspired by the aforementioned advantages, the multimetallic TMPs are widely studied; however, they often undergo phase separation, which prevents them from benefiting from the desired electronic configuration modulation. That makes the multimetallic phosphides difficult to design controllably.

Molybdenum phosphides (MoP) have recently been identified as a promising family of earth-abundant electrocatalysts, as Mo has the following benefits: (I) analogous electronic structure like Pt-group catalysts, which indicates a potential for high electrocatalytic activity.^26–28^ (II) DFT calculations suggest that the Gibbs free energy of hydrogen adsorption (Δ*G*_cat‑H*_) on MoP on selected facets is very low and suitable for HER.^29^ (III) The low cost and abundance of natural resources make them favorable for commercial applications.^30^ (IV) The generous design possibilities of the structure and morphology result in a high density of catalytically active sites. Scalability in the morphology lies in its large variety of structurally engineered morphologies, such as nanosheets,^31^ hollow particles,^32^ foams modified by MoP,^33^ nanotubes,^34^ and carbon substrates modified by MoP/MoSe.^35^ All of them offer a high degree of porosity in the microstructure for better access of the electrolyte to the active sites. However, the morphology critically depends on the preparation process and the reaction conditions. An important criterion when choosing a suitable method for the preparation of TMP is the transfer of results from the laboratory scale to industrially relevant production conditions (upscaling). Several multistep processes for the synthesis of phosphides are known, such as direct phosphorization of metal sources,^36^ decomposition of metal–organic precursors,^37^ gas–solid phase reactions,^38^ solid–state reactions,^39^ and solvothermal/hydrothermal methods.^40^ However, most of them have not gained commercial importance because they require expensive chemicals and specially designed reactors, produce highly toxic PH_3_, provide low yields, or are not suitable for continuous processes. Therefore, to increase its commercialization, it is very important to find a simple, cheap, fast, and nontoxic procedure where the evolution of highly toxic PH_3_ gas can be avoided.

This work introduces, for the first time, the synthesis of trimetallic phosphides (MoFeNiP and MoFeCoP) and their direct comparison with the bimetallic analogue MoFeP, using a nonconventional spray drying method (SDM) followed by optimized heat treatment. The SDM route provides a simple, low-cost, and scalable strategy for producing finely dispersed phosphide powders with tunable composition, uniform hollow-spherical morphology, and high surface area.^41^ Unlike conventional approaches, this method enables the rapid, large-scale preparation of multimetallic TMPs with nanoporous shells and without carbonaceous supports, ensuring abundant active sites, efficient mass transport, and enhanced electrocatalytic activity. To the best of our knowledge, this is the first demonstration of the fabrication of fine, hollow spherical trimetallic phosphide microparticles via spray drying, offering high activity, stability, and durability for future water electrolysis applications. An extraordinarily low activation energy, comparable to that of commercial noble-metal-based catalysts, was observed. This remarkable feature underscores the high intrinsic activity of the prepared trimetallic phosphides, highlighting their potential as cost-effective alternatives to noble-metal electrocatalysts for large-scale water electrolysis applications.

## 2 Experimental Section

### 2.1 Material Preparation

The source compounds for the preparation of phosphides included: ammonium phosphate dibasic ((NH_4_)_2_HPO_4_) (Centralchem, 99%), iron­(III) nitrate nonahydrate (Fe­(NO_3_)_3_·9H_2_O) (Centralchem, 98%), cobalt­(II) nitrate hexahydrate Co­(NO_3_)_2_·6H_2_O (Centralchem, 99%), nickel­(II) nitrate hexahydrate Ni­(NO_3_)_2_·6H_2_O (Centralchem, 98%), ammonium molybdate tetrahydrate (NH_4_)_6_Mo_7_O_24_·4H_2_O (Centralchem, 99%), and citric acid (CA) C_6_H_8_O_7_ (Centralchem, 99.5%). For electrochemical performance analysis, potassium hydroxide (KOH, Centralchem, 85%) was used as the electrolyte, and platinum on carbon (PtC, Sigma-Aldrich, *M*_w_ = 195.08 g·mol^–1^) and iridium oxide (IrO_2_, Sigma-Aldrich, *M*_w_ = 224.22 g·mol^–1^) were used as standard catalysts.

Precursors of MoFeP, MoFeNiP, and MoFeCoP hollow spherical particles were prepared by spray-drying an aqueous solution containing appropriate salts and CA. The input solution was prepared by gradually dissolving metal (Fe^III^; Mo^VI^;a and Ni^II^ or Co^II^) and phosphorus (P^V^) salts in a CA solution in amounts corresponding to the desired molar ratio in the final MoFeP, MoNiP, and MoCoP phosphides, CA/Fe/Mo/(Ni)/(Co)/P = 2:0.5:0.5:(0.5):(0.5):0.5. Specifically, 5.252 g of Fe­(NO_3_)_3_·9H_2_O, 2.295 g of (NH_4_)_2_Mo_7_O_24_·4H_2_O, 3.783 g of Co­(NO_3_)_2_·6H_2_O or 3.78 g of Ni­(NO_3_)_2_·6H_2_O, and 1.717 g of (NH_4_)_2_HPO_4_ were gradually dissolved in 250 mL of 0.4 M CA. After homogenization, a mixed solution was sucked into a spray dryer (TEFIC Biotech; TFS-2L) where the air drying was performed at an inlet air temperature of 300 °C (Figure S1). The solution was atomized by spraying droplets into a hot air stream. The drying conditions were chosen based on pilot experiments according to the residual water content in the spray-dried powder. The fan speed in the cyclone was set to 70% to prevent particle entrainment, a crucial step in ensuring that particles fall into the collecting jar. Additionally, the solution suction flow rate (controlled by the wiggle pump) was set to 30%, corresponding to a heating air flow rate of 0.83 mL min^–1^. The dried precursors of MoFeP, MoFeNiP, and MoFeCoP (depicted in Figure S2) were subsequently heat-treated at 650 °C under a reducing H_2_ atmosphere at a flow rate of 66 mL min^–1^.

### 2.2 Structural Methods and Characterization

The phase composition of the final spherical powder phosphides was identified by XRD analysis with a Cu Kα radiation source (PhilipsX′ PertPro) operating at 40 kV and 50 mA. The patterns were recorded at the 2 theta range between 10° and 60°.

The morphology and porosity of the sample were visualized by a scanning electron microscope (SEM, JEOL, JSM-7000F, Japan) equipped with an energy dispersive X-ray analyzer and a transmission electron microscope (TEM, JEOL, JEM-2100F, Japan) with high-resolution HR-TEM with selective diffraction area analysis (SAED). The thermal degradation of precursor samples was analyzed by differential thermogravimetric (DTG) analysis supplemented by thermal gravimetric (TG) analysis (NETZSCH STA449F3 Jupiter) at a heating rate of 10 °C·min^–1^ up to 1200 °C in an Al_2_O_3_ crucible under an air/argon atmosphere. The samples were processed under vacuum before analysis.

The chemical composition of the synthesized samples was determined by using X-ray photoelectron spectroscopy (XPS). XPS measurements were carried out using a custom-built spectrometer (SPECS Surface Nano Analysis GmbH, Germany) with a differentially pumped hemispherical electron analyzer operating under ultrahigh vacuum conditions (2 × 10^–9^ mbar). The system was equipped with a high-intensity, monochromated, microfocused Al Kα X-ray source (SPECS μ-FOCUS 600) and a multichannel electron energy analyzer (PHOIBOS 150 NAP 1D-DLD). Samples were pressed into pellets and mounted on a stainless-steel holder by using a stainless-steel strip. Low-resolution survey spectra were recorded with an acquisition step size of 1 eV (pass energy of 50 eV). Detailed XPS regions (Fe 2p, Mo 3d, Co 2p, Ni 2p, O 1s, C 1s, and P 2p) were acquired with an acquisition step size of 0.05 eV (pass energy of 20 eV). Deconvolution of detailed spectra was performed using KolXPD (1.8.0) software. A Shirley background and Voigt profiles were used for deconvolution. All 2p components were fitted with the ratio of p_3/2_/p_1/2_ peak intensities fixed at 2:1. Analogously, intensities of 3d_5/2_ and 3d_3/2_ peaks in 3d peaks were fixed at 3:2. The binding energies (BEs) were adjusted using the main adventitious sp^3^ carbon peak referenced at 284.9 eV, see Figure S4. The charging correction was in all cases about 1.1 eV, suggesting reasonable electronic conductivity of the samples.

### 2.3 Electrochemical Characterization

All electrochemical measurements were performed in a three-electrode setup in 1 M KOH using a Vionic potentiostat/galvanostat controlled by Autolab Intello software. A glassy carbon rotating disk electrode (GC RDE, Metrohm, Switzerland) with a diameter of 5 mm, modified with a thin layer of the prepared catalysts, was used as the working electrode. The revolution rate was maintained at 500 rpm for all measurements. An Ag/AgCl/3 M KCl electrode served as the reference electrode, while a platinum foil was used as the counter electrode. Pt RDE with 3 mm in diameter (Metrohm, Switzerland) was employed as a standard electrode for HER. The catalytic ink was prepared as follows: 750 μL of isopropanol (CentralChem, 99.7%), 250 μL of distilled water, 20 μL of Nafion (Nafion, perfluorinated resin solution, 5 wt % in lower aliphatic alcohols and water, contains 15–20% water, Aldrich), and 50 mg of catalyst were mixed. Then, 20 μL of the ink was dropped onto the GC electrode, corresponding to 1 mg of the catalyst on the GC surface (5.1 mg_catalyst_ cm^–2^). All potential values are recalculated with respect to a reversible hydrogen electrode (RHE) according to eq S1 provided in the Supporting Information. The electrochemical methods, including linear sweep voltammetry (LSV), cyclic voltammetry (CV), electrochemical impedance spectroscopy (EIS), and chronoamperometry, were utilized to evaluate the electrochemical performance of the prepared catalysts. All current densities (*j*) were calculated as a ratio of the recorded current and geometric surface area of the GC electrode (0.196 cm^2^). The reported potential values were recorded with 80% *iR* compensation performed during measurement. The EIS measurements were performed in the frequency range from 10 kHz to 0.1 Hz at an overpotential of −285 mV vs RHE with an applied potential amplitude of 5 mV. Spectra were fitted considering the equivalent circuits depicted in Figure S5 in the Supporting Information. The electrochemical surface area was compared based on CV measurement ±50 mV around the open circuit potential at different potential sweep rates (Figure S6A–D). The double-layer capacitance (*C*_dl_) was calculated by plotting Δ*j* (eq S4 in Supporting Information) against the scan rate. The chronopotentiometry measurement in 1 M KOH at a constant potential of −385 mV vs RHE for 22 h was used to evaluate the catalyst stability under HER operation. To evaluate the effect of temperature on HER performance, cathodic polarization was performed at a scan rate of 10 mV s^–1^ at temperatures from 298.15 to 338.15 K, with steps of 5 K. The apparent activation energy of HER at MoFeNiP and MoFeCoP is calculated according to eqs 7,8.

## 3 Results and Discussion

### 3.1 Structural and Morphological Characterization

The heat treatment procedure of phosphide precursors prepared by SDM was set according to differential thermal analysis (DTA) and TG analysis (the conditions are specified in detail in the Supporting Information). Figure 1 presents the TG profile of precursor samples recorded under an air/argon atmosphere (Figure 1A) and their corresponding DTA thermal behavior (Figure 1B). When discussing the hydrated forms of the input salts used (Fe­(NO_3_)_3_·9H_2_O; (NH_4_)_6_Mo_7_O_24_·4H_2_O; Co­(NO_3_)_2_·6H_2_O; or Ni­(NO_3_)_2_·6H_2_O), it is important to note that the SDM process was conducted at 300 °C, a temperature at which hydrates lose their weakly bound water. Therefore, the dried precursor samples exhibited strong hygroscopicity, allowing them to readily absorb moisture from the air. Consequently, the weight loss observed on TG curves in the interval from 100 to 250 °C may correspond not only to the conversion of hydrates to the anhydrous form but also to the content of nanocrystalline absorbed water in the spray-dried samples. The subsequent increase in temperature (from 250 to 650 °C) leads to the gradual decomposition of anhydrous salts through intermediate phases up to the formation of metal phosphates. Figure 1A illustrates that the thermal decomposition temperatures progress through successive stages, marked by sharp exothermic effects in DTA (Figure 1B). The temperatures at the peak maxima of the presented samples vary slightly depending on the inherent nature of the metal ions, following the trend: MoFeP at 470 °C, MoFeCoP at 567 °C, and MoFeNiP at 618 °C. According to these results, calcination above 650 °C is severe enough to cause the thermal decomposition of the used metal salts and (NH_4_)_2_HPO_4_, which was added as a phosphorus source, ultimately leading to the successful formation of phosphides in a reducing atmosphere of H_2_.

**Figure 1:**
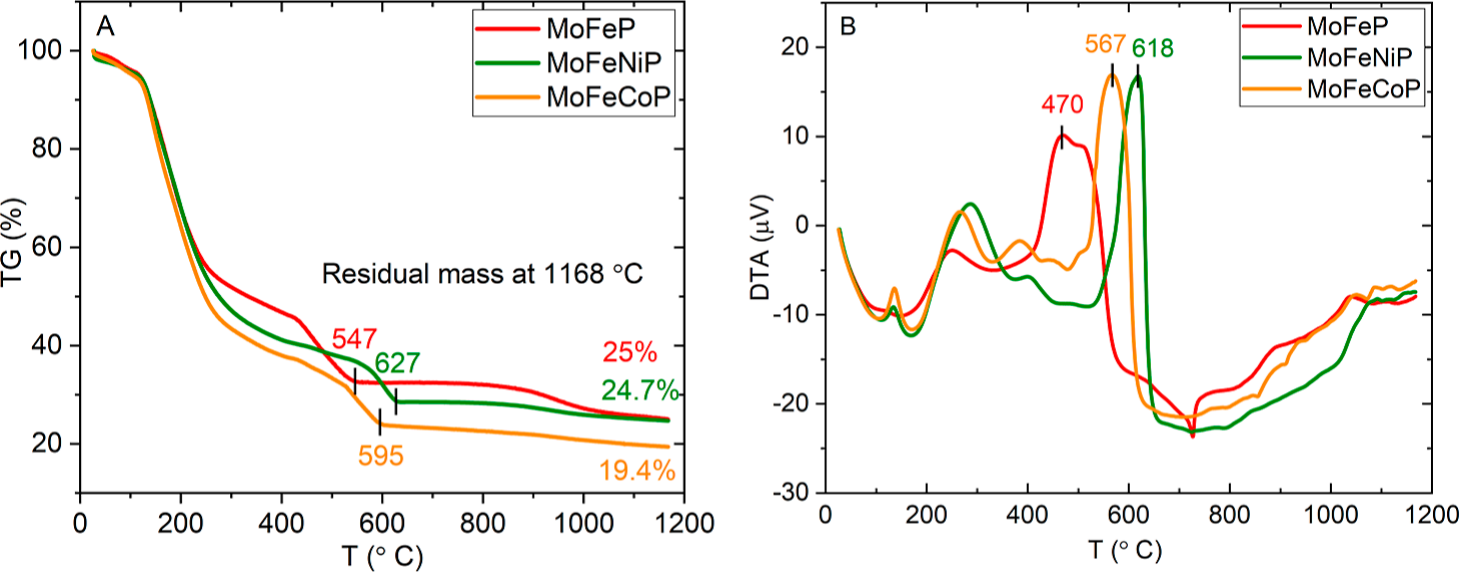
(A) TG analysis of MoFeP, MoFeNiP, and MoFeCoP and (B) DTA analysis of MoFeP, MoFeNiP, and MoFeCoP.

The three phosphides (MoFeP, MoFeNiP, and MoFeCoP) synthesized at 650 °C in a reducing atmosphere were characterized by using XRD analysis (Figure 2). The bulk crystal structure of all identified samples showed identical reflections, indicating that Ni and Co were successfully incorporated into the orthorhombic structure of MoFeP with space group *Pnma*, in accordance with ICDD 04-001-4637 (*a* = 5.922 Å, *b* = 3.663 Å, and *c* = 6.79 Å). The most intense peak at 37.9° (321) plane, in comparison with the peak at 42.3° (411), confirms the presence of a thermodynamically stable facet of the (321) plane. Based on the XRD results, the crystal structure was further confirmed by TEM and HR-TEM analysis to examine the nanoscale crystal structure of the catalysts and determine whether the bulk crystal structure was representative of the material throughout the individual crystals. TEM observations confirmed the results obtained from the XRD analysis. Figure 3 presents the acquired images. The first row shows the morphologies of the particles at lower magnifications. It is important to note that the presented images were not captured at the same magnifications; therefore, for comparison, the individual scale bars should be carefully considered. The images reveal the globular nature of the particles. The second row displays selected regions of the particles in the HR-TEM imaging. The visible crystalline planes or atom columns provide clear evidence of the crystalline nature of the particles. A compelling confirmation of crystallinity is further supported by the fast Fourier transformation (FFT) patterns. The measured and identified frequency spots in the FFT patterns correspond to the diffraction reflections of the orthorhombic structure identified by the XRD analysis. The low-intensity XRD peak observed at approximately 38.44° in the MoFeP sample (red curve in Figure 2) can be attributed to the (321) plane of a tetragonal crystal system with the *I*4̅2*m* space group. According to the JCPDF reference pattern 01-089-2587, this peak likely corresponds to a crystalline phase associated with a secondary minority phase, identified as the metal phosphide Mo_3_P.

**Figure 2:**
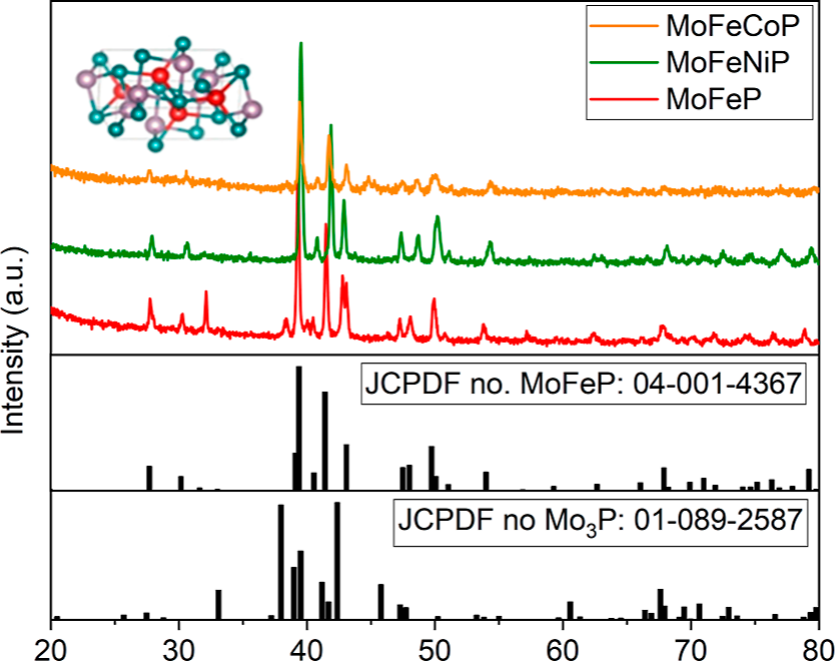
XRD pattern of MoFeP, MoFeCoP, and MoFeNiP.

**Figure 3:**
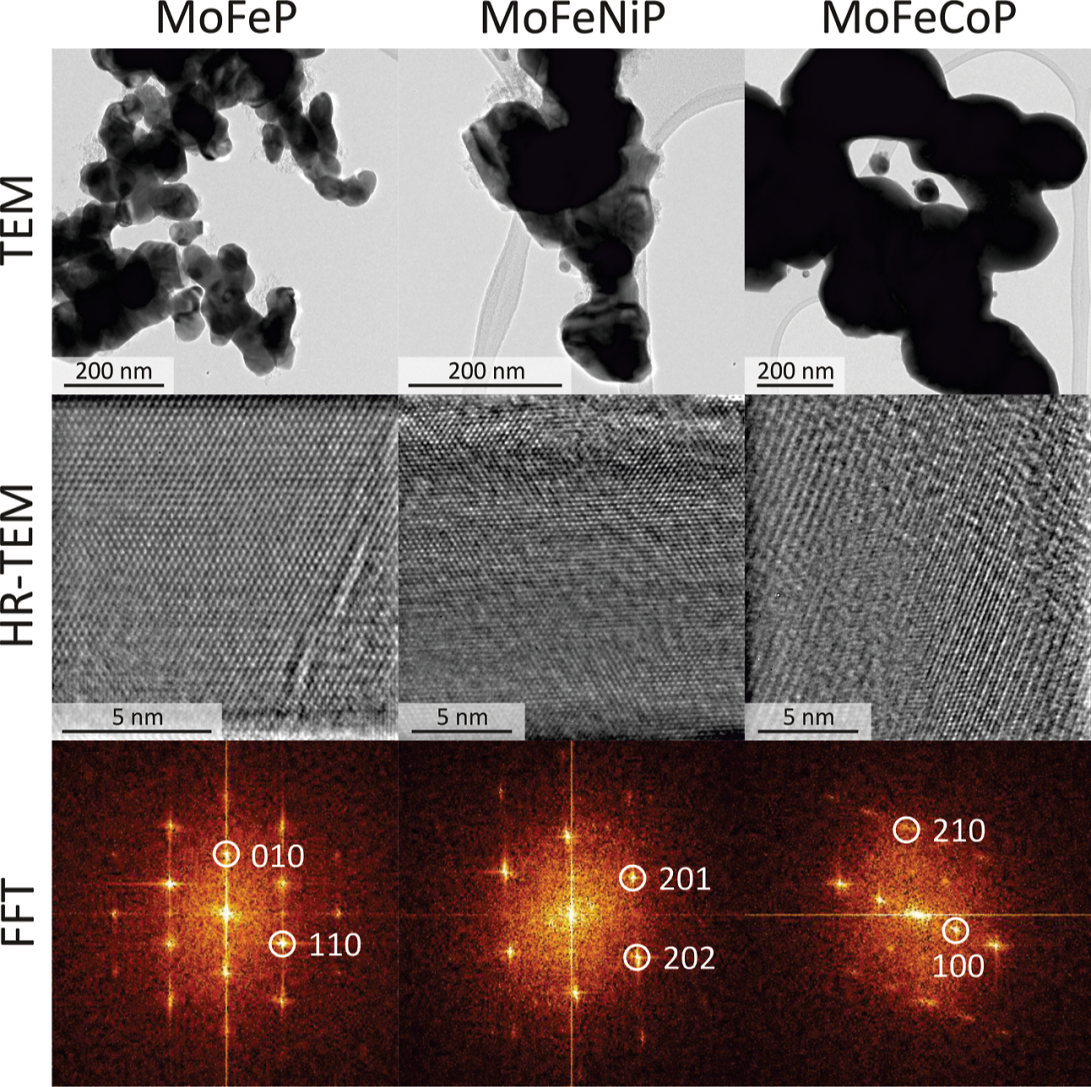
Structure of individual compositions as observed by TEM. The first row presents micrographs with low magnification, allowing a comparison of particle morphology. The second and third rows display HRTEM and FFT images of the structures, respectively, providing evidence of the crystalline nature of the particles.

The SEM images in Figure 4A–C obtained immediately after the SDM show the regular spherical powder structure with a hollow character and brittle shell. The spheres possess a wide distribution of diameters ranging from 5 to 10 μm. The SDM formed amorphous spherical particles with a very smooth surface. However, it is quite obvious from the comparison of Figure 4A–C with Figure 4D–F, showing the powders after heating in H_2_ at 650 °C, that the heat treatment in the reduction atmosphere led to a significant increase of porosity, though the spherical morphology was maintained, including the presence of the hollow part of the particles. However, the particle diameter was approximately halved, while the shell thickness increased. The morphology of the resulting MoFeP, MoFeNiP, and MoFeCoP particles consisted of rounded, irregular particles joined together to form spherical hollow structures, creating a regular interstructural porosity in the shell.

**Figure 4:**
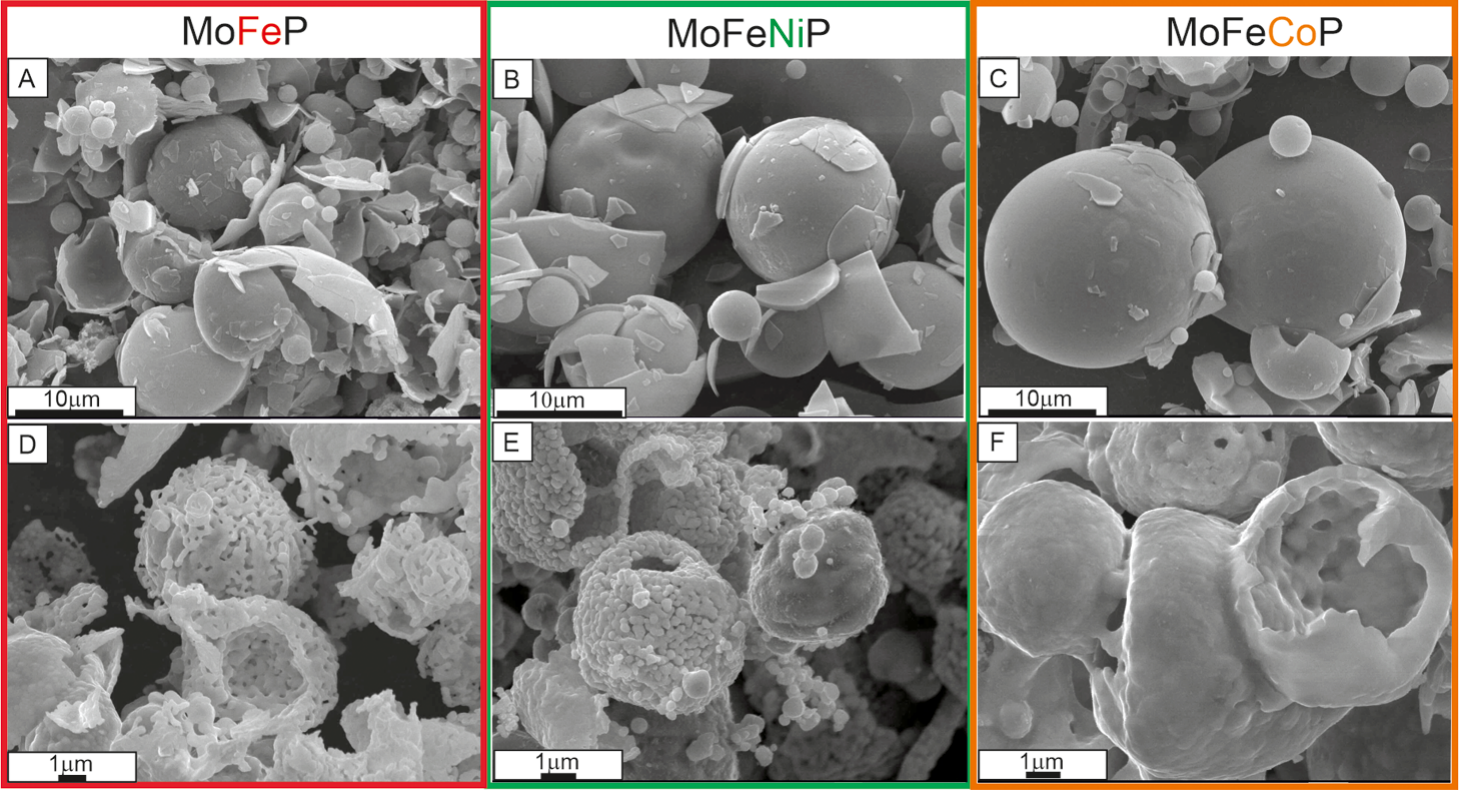
SEM images of powdered spherical samples: (A–C) immediately after the SDM method and (D–F) after sintering at 650 °C in a H_2_ atmosphere.

### 3.2 XPS Analysis

The high-resolution XPS spectra of Mo 3d, Fe 2p, P 2p, Co 2p, and Ni 2p regions acquired from the prepared catalysts are shown in Figures 5 and 6. Survey spectra of the samples are presented in Figure S3 of the Supporting Information. They confirm the presence of all expected elements. Mo 3d spectra of all investigated samples could be reasonably well fitted with three doublets; see Figure 5. It is clear that the introduction of both Co and Ni into the MoFeP structure causes a slight shift (0.2–0.3 eV) of all Mo 3d bands to lower BE. Except for this shift, Mo 3d spectra are almost identical for all samples. Therefore, it is sufficient to discuss in detail only the Mo 3d spectrum of MoFeP. The asymmetric doublet with the Mo 3d_5/2_ peak at about 228.4 eV and the corresponding asymmetric Mo 3d_3/2_ component at about 231.6 eV (with spin–orbit splitting, i.e., ΔBE_228_ ≈ 3.2 eV) suggests the presence of Mo phosphides.^42^ The two symmetric doublets with Mo 3d_5/2_ peaks at higher energies (229.4 and 232.9 eV), both with ΔBE ≈ 3.17 eV, can be attributed to Mo^4+^ and Mo^6+^ oxides.^43^ The corresponding Fe 2p spectra could have been fitted in multiple ways. The one well-defined and easily identifiable feature in all Fe 2p spectra is the doublet of iron phosphide with Fe 2p_3/2_ at 707.5 eV (ΔBE_707_ ≈ 12.9 eV) for all the samples.^44^ The rest of the spectra could be deconvoluted into two very broad peaks or several thinner ones with Fe 2p_3/2_ around 712 eV, corresponding to FeO_*x*_.^45^ Due to the unambiguity of the fit, the individual peaks are not presented. P 2p doublets in all samples are also analogous. There is a pronounced doublet with P 2p_3/2_ line at about 130.0 eV (ΔBE_707_ ≈ 0.88 eV) corresponding to metal phosphides.^46^ The second feature is a broad doublet of phosphates with a P 2p_3/2_ binding energy of about 133.6 eV for MoFeP (133.3 eV for MoFeNiP and MoFeCoP) with ΔBE_133_ ≈ 0.84 eV.^47^

**Figure 5:**
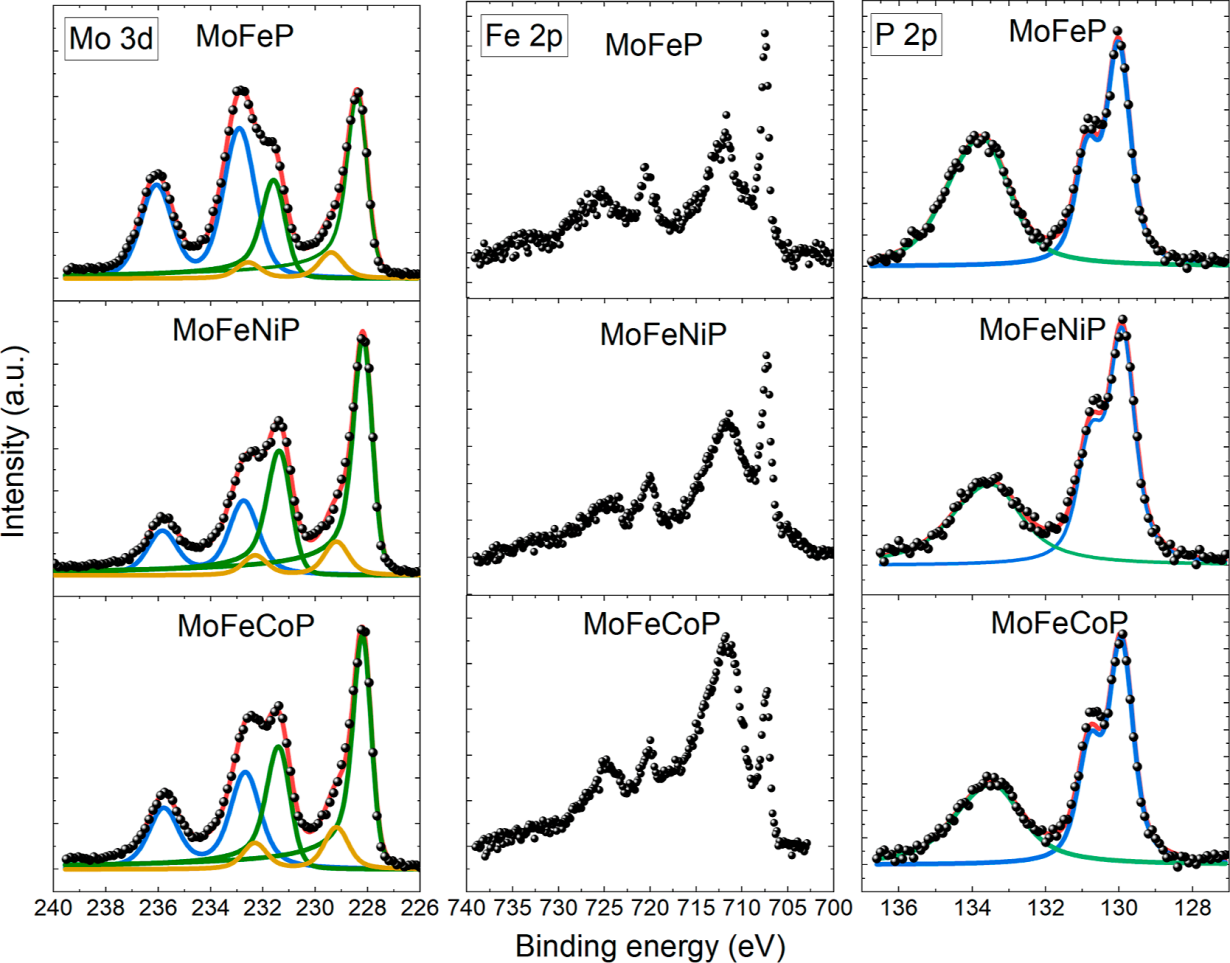
Detailed photoelectron spectra of Mo 3d, Fe 2p, and P 2p core levels in MoFeP, MoFeNiP, and MoFeCoP. Mo 3d and P 2p spectra were Shirley background corrected and deconvoluted, Fe 2p only Shirley background corrected.

The Co 2p spectrum of MoFeCoP was fitted using three peak doublets, see Figure 6A. In particular, the narrow doublet with 2p_3/2_ at 778.9 eV (ΔBE_778_ ≈ 14.9 eV) confirms the presence of phosphide in the surface layer.^42^ Another two Co 2p doublets (with 2p_3/2_) at 781.2 and 786.0 eV (with ΔBE_781_ ≈ 15.9 eV and ΔBE_786_ ≈ 18.1 eV) correspond to the core Co levels in phosphate and the associated satellite, respectively.^48^ Except for a binding energy shift, the presented Co 2p spectrum of MoFeCoP agrees well with that determined in our previous work for NiCoP.^18^ It must also be mentioned that while the presented fit is only approximate, it is sufficient to confirm the presence of phosphide in the surface layer. Appropriate fit would require a thorough analysis of Auger peaks, which overlap the Co 2p_3/2_ region.^49^

**Figure 6:**
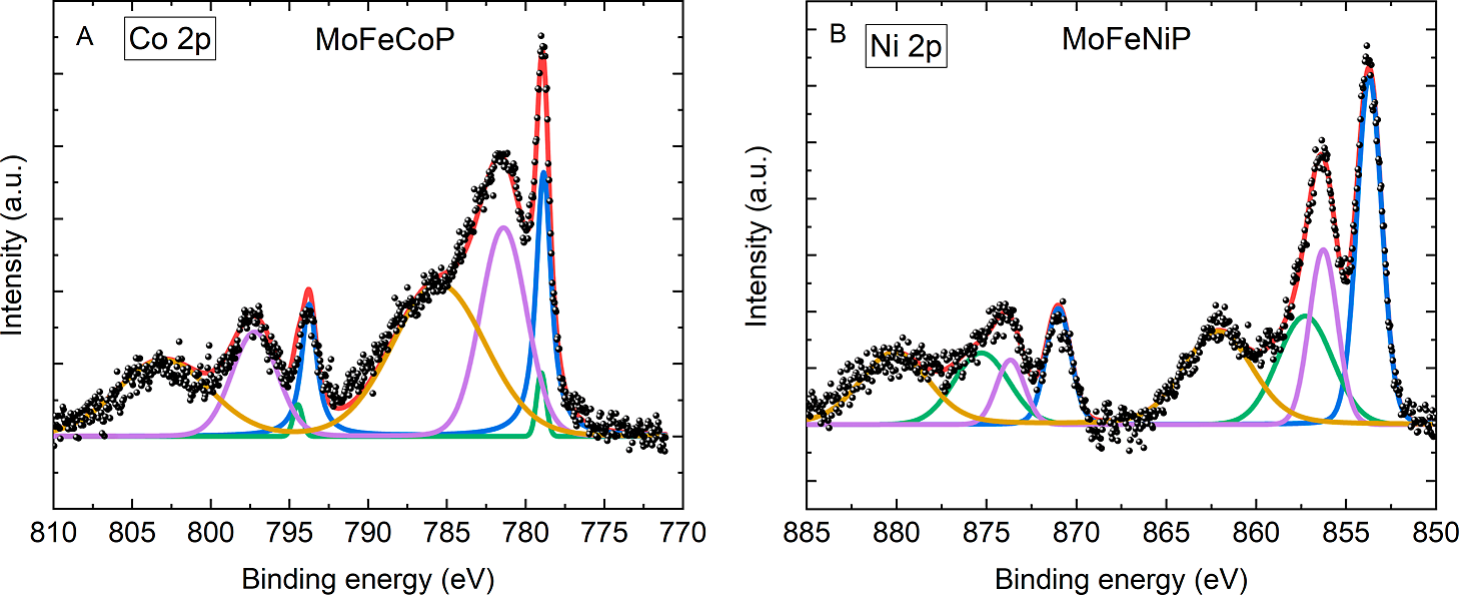
Detailed photoelectron spectra of (A) Co 2p and (B) Ni 2p core levels in MoFeNiP and MoFeCoP, respectively. Spectra were Shirley background corrected and deconvoluted.

The Ni 2p spectrum of MoFeNiP was deconvoluted into four doublets; see Figure 6B. The doublet at the lowest energy (Ni 2p_3/2_ at 853.7 eV, ΔBE_853_ ≈ 17.3 eV) corresponds most likely to phosphide. Ni 2p_3/2_ peaks at 856.3 eV (ΔBE_856_ ≈ 17.4 eV) and 857.3 eV (ΔBE_857_ ≈ 18.0 eV) suggest the presence of Ni^2+^ surrounded by sufficiently electronegative atoms such as O, corresponding to a partly oxidized surface layer and consistent with the presence of phosphates in the surface layer. Finally, the doublet with Ni 2p_3/2_ at 862.1 eV (ΔBE_862_ ≈ 18.0 eV) corresponds to satellite peaks of Ni^2+^.

### 3.3 Electrochemical Characterization

#### 3.3.1 HER Activity in 1 M KOH Electrolyte Solution

The electrocatalytic performance and operational stability of the synthesized catalysts toward the HER in a 1 M KOH environment were systematically investigated by using LSV and EIS analysis (Figure 7). The HER activity of the phosphide-based catalysts was compared with that of the bare GC electrode and a commercial Pt electrode. It is worth noting that some studies of phosphide-based HER catalysts in an alkaline environment employ commercial Pt/C (with a higher surface area ensuring catalytic activity) as the HER benchmark catalyst.^50,51^ Compared to a Pt/C-based thin layer at RDE, the RDE with a bulk Pt disk utilized in the present work provides better-defined geometry but also a lower surface area, resulting potentially in slightly higher overpotentials compared to Pt/C-based benchmarks. Values of overpotentials at various current densities are summarized in Figure 5A and Table S1 in the Supporting Information. As expected, the bare GC electrode exhibited a very high overpotential (η_–10_ = −718 mV). On the other hand, the bulk Pt electrode with η_–10_ = −100 mV displayed superior HER activity among all tested samples. As shown in Figure 7A,B, the η_–10_ values for the prepared catalysts increased in the order of MoFeCoP (−285 mV) < MoFeP (−337 mV) < MoFeNiP (−421 mV), indicating that Co incorporation into the MoFeP structure leads to a more active catalyst. On the contrary, Ni seems to reduce catalytic activity.

**Figure 7:**
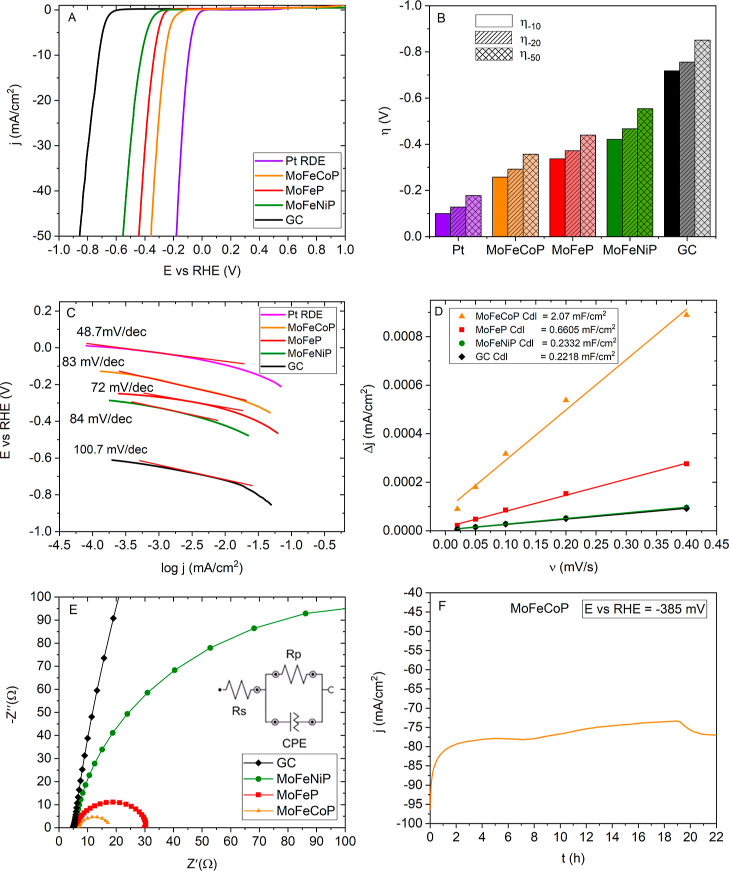
Electrochemical analysis of MoFeP, MoFeNiP, and MoFeCoP spheres in 1 M KOH (A) LSV, (B) η_–10_, η_–20_, and η_–50_, (C) Tafel plots, (D) capacitive currents as a function of different scan rates with *C*_dl_ data, (E) Nyquist plots of impedance spectra at overpotential of −285 mV, and (F) chronoamperometric stability test (*I*–*t*) of MoFeCoP at −385 mV vs RHE for 22 h at 500 rpm.

Possible steps of HER on the investigated catalysts can be described by eqs 4–7. The Tafel analysis (Figure 7C and Table S1 in Supporting Information) revealed comparable Tafel slope values for all prepared samples, MoFeCoP (−83 mV dec^–1^), MoFeP (−72 mV dec^–1^), and MoFeNiP (−84 mV dec^–1^), indicating that the rate-determining step in the HER mechanism corresponds to electrochemical desorption, specifically the Heyrovsky reaction (eq 6). If this is the case, it suggests relatively strong M–H binding, resulting in slow H desorption. Therefore, fine-tuning the catalyst composition in order to lower the Gibbs adsorption energy of hydrogen could offer an avenue for potentially enhancing HER kinetics.

4 $$Volmer\;step:\;MoFeP^{*}+H_{2}O+e^{-}\to MoFePH^{*}+OH^{-}$$

5 $$\text{and}\;Heyrovsky\;step:\;MoFePH^{*}+H_{2}O+e^{-}\to MoFeP^{*}+OH^{-}+H_{2}$$

6 $$\text{or}\;Tafel\;step:\;MoFePH^{*}+MoFePH^{*}\to 2MoFeP^{*}+H_{2}$$

The analysis (Figure 7E) of EIS recorded at an HER overpotential of −0.285 V provided similar results concerning the charge transfer properties of the synthesized catalysts. As expected, the Ohmic resistance (*R*_s_) values (Table 1) were relatively similar across all samples. However, significant variations were observed in the charge transfer resistance (*R*_p_), which reflects the ease of electron transfer during HER at the electrode–electrolyte interface. The MoFeCoP demonstrated the lowest *R*_p_ (11.7 Ω), followed by MoFeP (25.2 Ω) and MoFeNiP (215 Ω). The notably low *R*_p_ of MoFeCoP suggests enhanced electron transport and faster reaction kinetics, which correlate with its superior HER performance observed by LSV. There are two possibilities for explaining these results: either differences in electrocatalytic activity or electrochemically active surface area of the catalysts (or a combination of both). To answer this question, it is valuable to explore the double-layer capacitances *C*_dl_ of the catalysts in a thin layer (Figure 7D), as a measure of electrochemically active surface area, determined by CV measurements in nonfaradaic regions (Figure S6). These values increase in order MoFeNiP (0.23 mF cm^–2^) < MoFeP (0.66 mF cm^–2^) < MoFeCoP (2.07 mF cm^–2^). Very similar are the values of effective differential capacitance *C*_eff_ calculated from the impedance data in Table 1 according to eq S5. Results are summarized in Table 1. Consequently, the high activity of MoFeCoP can be attributed to its high electrochemical surface area. The “1/*R*_p_” values (proportional to the rate of HER at a given overpotential) can be normalized by the *C*_eff_ values (proportional to the electrochemically active surface), resulting to “1/(*R*_p_·*C*_eff_)”. Since the as-obtained values are very similar for all three investigated catalysts, it seems that they possess active sites of very similar HER activity.

**Table 1:** Values of *R*_s_, *R*_p_, Constant Phase Element (CPE) Parameters (*Y*_0_ and α), and Χ^2^ (an Error in EIS Fit) for All Samples Obtained by Fitting HER Impedance Spectra in an Alkaline Environment with an Equivalent Circuit^a^

			CPE^b^			
sample	*R*_s_ [Ω]	*R*_p_ [Ω]	*Y*_0_ [μS s^α^]	α	Χ^2^	*C*_eff_ [mF cm^–2^]
MoFeCoP	6.60	11.7	743	0.874	0.0297	1.87
MoFeP	5.36	25.2	181	0.917	0.093	0.56
MoFeNiP	5.25	215	63.1	0.928	0.0129	0.23
GCE	4.93	7230	108	0.881	0.0811	0.52

a *C*_eff_ is the effective differential capacitance, calculated using eq S5.

b CPE in EIS experiments is described in the Supporting Information in detail.

Although the prepared materials do not reach the ultralow overpotentials of some benchmark catalysts listed in Table S3, their unique advantages should be emphasized. These carbon-free microparticles are exceptionally easy to process into catalytic layers for electrolyzers and fuel cells, offering clear benefits for practical and commercial applications. Moreover, the synthesis route is considerably simpler, faster, and far more suitable for large-scale production than most reported methods, positioning these materials among highly promising candidates for real-world electrochemical energy technologies.

To assess the long-term durability, chronoamperometry measurements were conducted at a constant potential of −385 mV vs RHE for 22 h on the most active catalytic sample, MoFeCoP (Figure 7F), and also for other two investigated catalysts, i.e. MoFeP and MoFeNiP (Figure S7). As depicted in Figure 7F, the catalyst exhibited a moderate current attenuation over time, indicating reasonable stability under continuous operation. Increase in the current density after prolonged HER operation is likely related to an electrochemical activation process. Such behavior is commonly observed in TMP-based catalysts, where continuous operation can lead to restructuring of the surface, improved electrode wettability, or exposure of new active sites, all contributing to enhanced catalytic activity.

The HER activity of MoFeP, MoFeNiP, and MoFeCoP catalysts in alkaline media can be attributed to a synergistic effect between Mo, Fe, and the additional 3d transition metals (Ni or Co) within the phosphide matrix. Mo sites are known to facilitate the adsorption and activation of water molecules, thereby accelerating the Volmer step,^52^ while Fe and the other metals (Ni or Co) modulate the electronic structure, optimizing hydrogen adsorption free energy (delta Δ*G*_H_*). The incorporation of P enhances the intrinsic conductivity and provides electron-rich sites to further stabilize adsorbed H intermediates.

Prolonged operation can lead to the partial transformation of the phosphide surface into catalytically active amorphous oxy/hydroxide or phosphate phases, as commonly observed in TMPs.^53^ This process can expose more active sites and improve the catalytic durability. Incorporation of multiple metals may offer additional stabilization via the formation of mixed-metal phosphate layers, known to be robust under alkaline HER conditions. These dynamic changes contribute to both the high activity and long-term operational stability observed in our catalysts.

Finally, the effect of the temperature on the catalytic activity of MoFeCoP and MoFeNiP was also evaluated. The LSV curves recorded at various temperatures are presented in Figure S8A,B. In order to compare currents measured at different temperatures at the same overpotentials, the electrode potentials measured with respect to *E*_Ag/AgCl_ (Ag/AgCl/3 mol/L of KCl) were recalculated to the RHE scale. This involves considering the potential shift of reference electrode *E*_Ag/AgCl_ (Ag/AgCl/3 mol/L KCl) due to temperature variation; see eq S6 and Table S2 in the Supporting Information.

For both electrodes, a pronounced temperature effect on the HER activity was observed. With a temperature increase of 35 K and at an overvoltage of −500 mV, the MoFeCoP catalyst achieved an increase in current density of −195 mA cm^–2^ (from −145 mA cm^–2^ to −340 mA cm^–2^), which is superior when compared to an increase of 87 mA cm^–2^ (from −58 mA cm^–2^ to −145 mA cm^–2^) for MoFeNiP (Figure S8A,B). For both phosphide samples, Tafel plots were constructed to estimate the exchange current densities (*j*_0_) at zero overpotential, as shown in Figure S8C,D in the Supporting Information. The plots of ln *j*_0_ versus 1/*T* exhibit a linear Arrhenius-like dependence, as described by eqs 7 and 8. From the dependence, an apparent activation energy, *E*_a_, can be calculated.

7 $$j_{0}=J_{S}e^{\frac{-E_{a}}{RT}}$$

8 $$\ln j_{0}=\ln J_{S}-\frac{E_{a}}{R}\frac{1}{T}$$

where *J*_S_ stands for “a current density pre-exponential factor”, *R* is the universal gas constant (*R* = 8.314 J K^–1^ mol^–1^), and *T* is the absolute temperature. From the relevant dependence, the apparent activation energy *E*_a_ can be readily determined. The slope of semilogarithmic plots of ln *j*_0_ versus the inverse temperature 1/*T* shown in Figure 8A,B yielded *E*_a *j*0_ values of 24 kJ mol^–1^ for MoFeNiP and 21 kJ mol^–1^ for MoFeCoP. The similar *E*_a_ values were also found for both samples at the overpotential of −0.5 V (ln *j*_–0.5 V_ vs 1/*T*), where the reaction proceeds efficiently, as shown in Figure 8C,D. The corresponding *E*_a,–0.5 V_ values were 23 kJ mol^–1^ for MoFeNiP and 19 kJ mol^–1^ for MoFeCoP. These *E*_a_ values are rather low for TMP-based HER catalysts, which typically exhibit *E*_a_ of around 50 kJ mol^–1^ depending on their composition, synthesis method, intrinsic conductivity, and electronic structure^54^ and are close to the *E*_a_ values for HER on Pt (∼20 kJ mol^–1^).^55^ However, the proper tuning of TMP through the combination of Ni, Mo, and Mo, Co along with the creation of multimetallic phosphides can utilize synergistic effects, which can play a crucial role in enhancing electrocatalytic performance for HER and reducing the value of the activation energy *E*_a_.^56,57^ For example, Xia et al. achieved an *E*_a_ of 21 kJ mol^–1^ in the trimetallic Ni–Mo–Cu compared to the *E*_a_ of 36 kJ mol^–1^ for the bimetallic Ni–Mo.^58^ The reduction in the *E*_a_ value was attributed to the incorporation of Cu into the metallic structure, which led to an increase in surface area, the number of active sites, and synergistic interactions between Ni, Mo, and Cu, compared to Ni–Mo. Tn the catalyst electronic structure, as demonstrated by DFT calculations. This synergy optimizes the active site density and catalyst electronic conductivity, thereby promoting an accelerated HER rate. Similar low *E*_a_ values were observed for Ni-based and NiCr porous electrodes at different cathodic overpotentials (from −50 mV to −300 mV) using potentiodynamic tests .^59^ The results showed that the *E*_a_ was lower for the Ni-based system at equilibrium; however, the NiCr porous electrode exhibited better performance due to its lower values of *E*_a_. In the work of Wu et al., the *E*_a_ for the porous Ni–Cr–Fe electrode in simulated seawater was studied as calculated from the slope of log *j*_0_ versus 1/*T*. It was found that the *E*_a_ of the porous Ni–Cr–Fe electrode, as a ternary metal system, was only 28 kJ mol^–1^, i.e., lower than that of pure Ni (35 kJ mol^–1^), Fe (39 kJ mol^–1^), and the Ni–Fe binary alloy (31 kJ mol^–1^) in alkaline solution. Although these comparisons are based on different environments, due to the limited availability of *E*_a_ data for similar systems in simulated seawater, the porous Ni–Cr–Fe electrode appears to exhibit higher catalytic activity by lowering the activation energy of the reaction.

**Figure 8:**
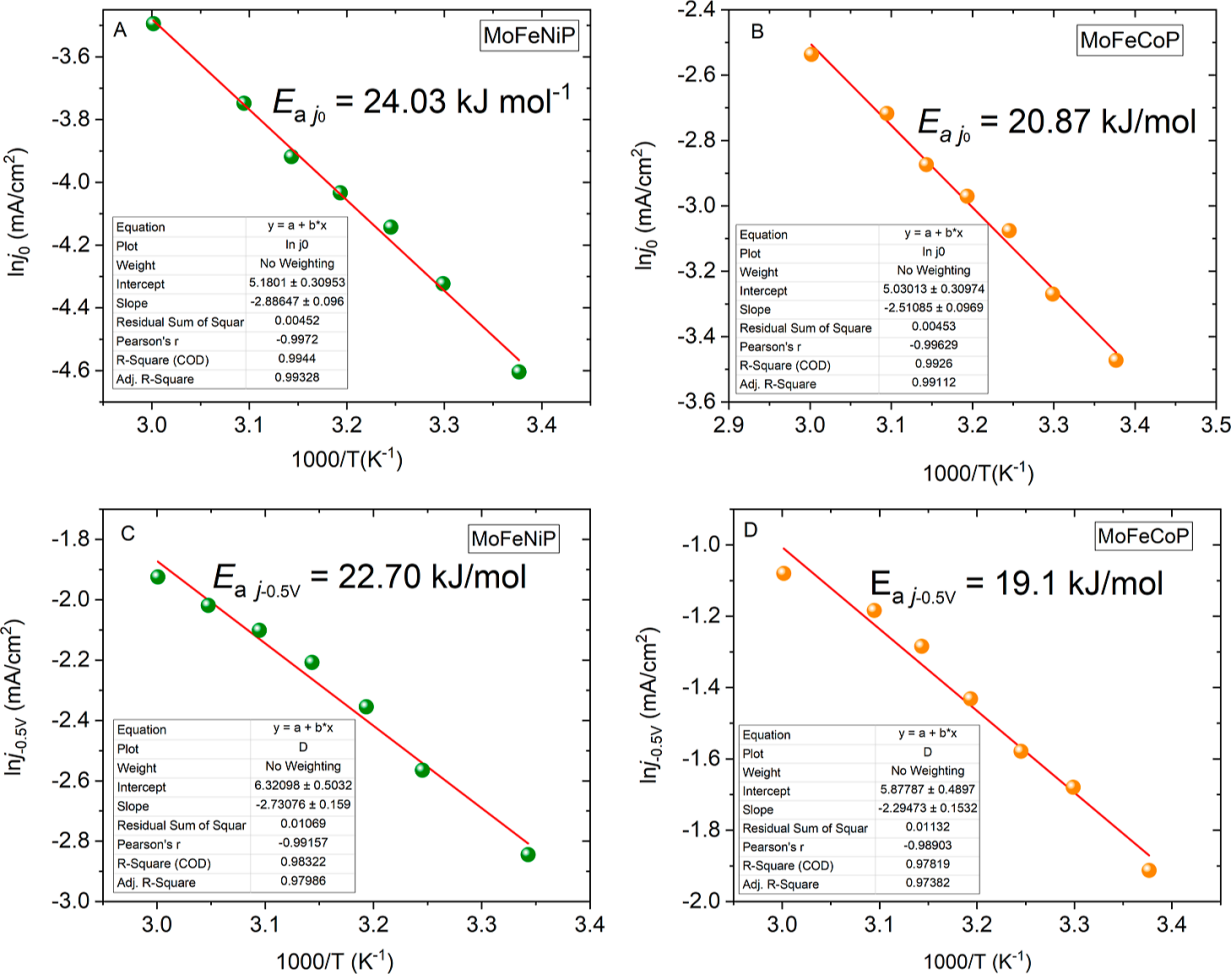
Arrhenius plots for MoFeNiP (A,C) and MoFeCoP (B,D) determined at equilibrium potential of HER, i.e., calculated from *j*_0_ values (A,B), and HER overpotential of −0.5 V, i.e., calculated at *j*_–0.5 V_ (C,D).

## 4 Conclusion

This study reports the scalable, user-friendly, and low-cost preparation of innovative spherical phosphide powders based on transition metals, namely, MoFeP, MoFeNiP, and MoFeCoP. The facile and cost-efficient SDM was used to produce hollow spherical precursor particles on a large scale, followed by a heat treatment process at 650 °C in a reducing atmosphere for the final formation of spherical phosphides. The prepared spheres, with their unique 3D structures, serve as examples of catalysts for the HER in the AWE process, wherein MoFeCoP has been proven to be a highly efficient non-noble electrocatalyst.

The electrocatalytic performance of MoFe-based phosphides toward the HER in an alkaline environment was systematically evaluated. Among the synthesized catalysts, MoFeCoP exhibited the highest HER activity, achieving the overpotential (at −10 mA cm^–2^) of η_–10_ = −285 mV and significantly surpassing both MoFeP (η_–10_ ≈ −337 mV) as well as MoFeNiP (η_–10_ ≈ −421 mV). The Tafel slope analysis confirmed that HER on all phosphide catalysts followed the Volmer–Heyrovsky mechanism, with the Heyrovsky reaction being the rate-determining step. MoFeCoP demonstrated the most favorable HER kinetics. The superior catalytic activity of MoFeCoP was attributed to its enhanced electroactive surface area and reduced charge transfer resistance, as revealed by double-layer capacitance and EIS. Moreover, chronoamperometry measurements confirmed its reasonable stability during 22 h of continuous operation at −385 mV versus RHE. These findings highlight the potential of Co-doped phosphides as efficient electrocatalysts for alkaline water splitting applications, providing valuable insights for future catalyst design and optimization. The use of cost-effective, abundant, and easy-to-synthesize TMP as alternatives to noble metals further supports their widespread commercial applications and enables hydrogen production on a scale capable of meeting global energy demand. Thus, this highly innovative approach to catalyst preparation, along with the resulting unique catalyst form produced on a large scale, opens new pathways for the production and application of TMP-based catalysts, as well as other alternatives for future commercial applications.

## Supplementary Material



## Data Availability

You can cite all versions by using the DOI http://doi.org/10.5281/zenodo.15657026. This DOI represents all versions, and will always resolve to the latest one. Data set: http://doi.org/10.5281/zenodo.15657027. Cite all versions? You can cite all versions by using the DOI http://doi.org/10.5281/zenodo.15657042. This DOI represents all versions, and will always resolve to the latest one. Preprint: http://doi.org/10.5281/zenodo.15657043.

## Notes

Preparation of phosphide precursors; MoFeP, MoFeNiP, and MoFeCoP powder precursors after spray drying; X-ray photoelectron spectra of investigated samples; detailed X-ray photoelectron C1s spectra of investigated samples with deconvolution photographs of the experimental setup; values of overpotentials at current densities; circuits used for fitting EIS measurements in 1M KOH; standard potential of reference *ET* Ag/AgCl/3 M KCl electrode vs RHE with increasing temperature; chronoamperometric stability test; LSV curves; and comparison of the HER performance of multimetallic TMPs with recent literature reports (PDF)
