# Supplementary material for: Synthesis of Hollow Spherical Phosphide Catalysts with Industrial-Scale Potential for Alkaline Hydrogen Evolution Reaction
Source: ACS Appl Mater Interfaces. 2025 Sep 22;17(39):54749–60. doi: 10.1021/acsami.5c11704 (PMC12492331; doi:10.1021/acsami.5c11704)
Supplement: Supplementary file 1 [file am5c11704_si_001.pdf]

Supporting Information

Synthesis of Hollow Spherical Phosphide Catalysts with Industrial-Scale Potential for Alkaline Hydrogen Evolution Reaction

Magdalena Streckova [a*], Alena Fedorockova [b], Alexandra Guboova [a], Gabriel Sučik [b], Vladimir Girman [a,c], Akbar Hussain [d,e], Michael Vorochta [f], Jozef Strečka [g], Tomas Bystron[h*]

[a] *Institute of Materials Research, Slovak Academy of Sciences, Watsonova 47, 040 01, Kosice, Slovak Republic.*

[b] *Faculty of Materials, Metallurgy and Recycling, Technical University of Kosice, Letna 9, 042 00, Kosice, Slovakia.*

[c] *Institute of Physics, Faculty of Science, P.J. Safarik University, Park Angelinum 9, 041 01 Kosice, Slovak Republic*

[d] *Department of Chemistry, Quaid-i-Azam University, Islamabad, 45320, Pakistan*

[e] *Department of Physical Chemistry, Faculty of Science, P.J. Šafárik University, Moyzesova 11, SK-04154 Košice, Slovak Republic*

[f] *Department of Surface and Plasma Science, Faculty of Mathematics and Physics, Charles University, V Holešovičkách 2, 18000 Prague 8, Czech Republic.*

[g] *Department of Theoretical Physics and Astrophysics, Faculty of Science, P.J. Šafárik University, Park Angelinum 9, 040 01 Košice, Slovak Republic*

[h] *University of Chemistry and Technology Prague, Department of Inorganic Technology, Technicka 5, Prague 6, 166 28, Czech Republic*

**E-mail: bystront@vscht.cz*

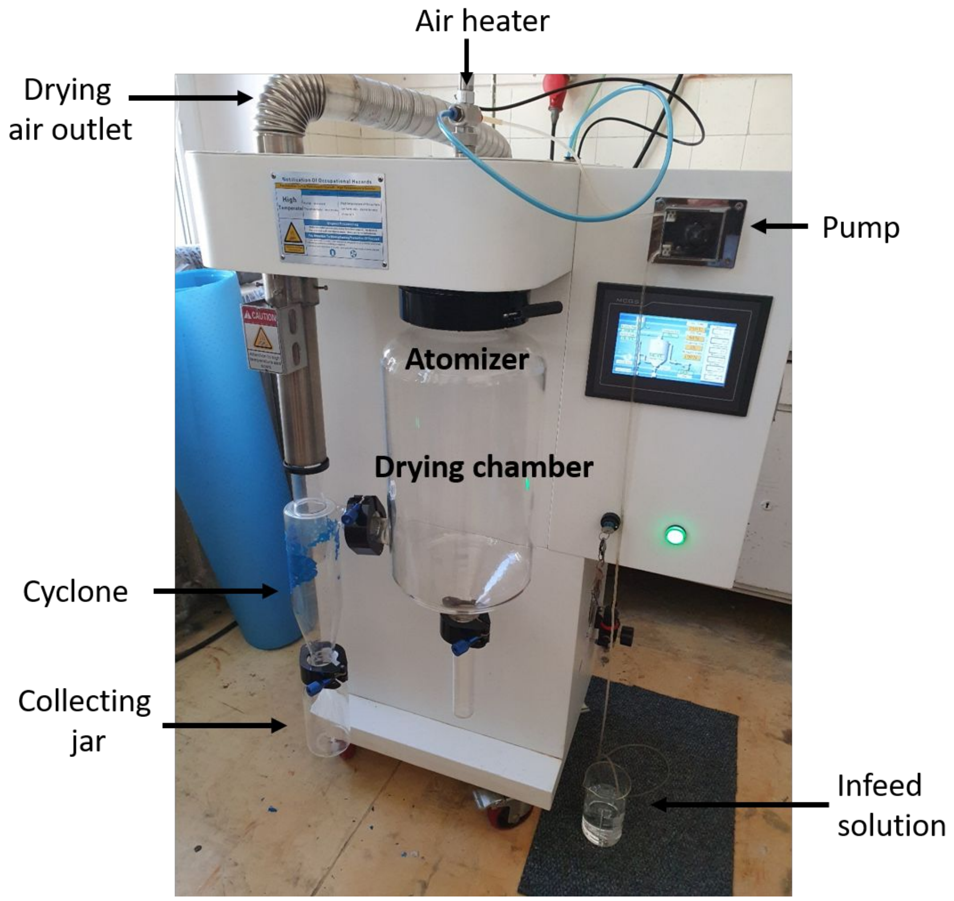

Figure S1 Drying device TEFIC Biotech; TFS-2L used for the preparation of phosphide precursors

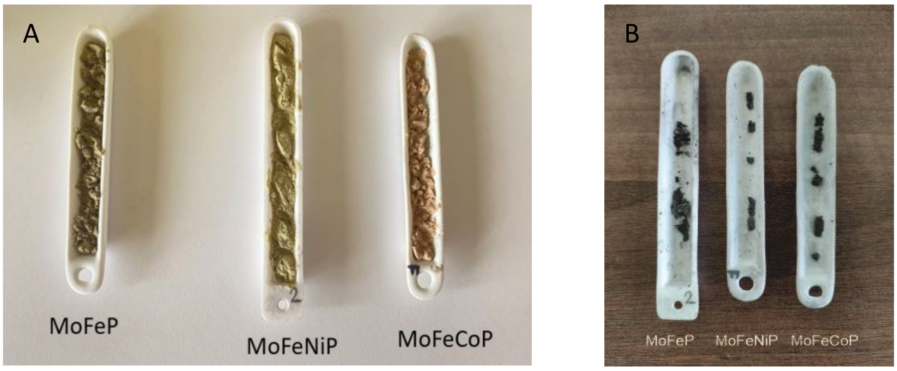

Figure S2 MoFeP, MoFeNiP and MoFeCoP (A) powder precursors after spray drying (B) powders after calcination in a reducing atmosphere of H_2 at 650 °C

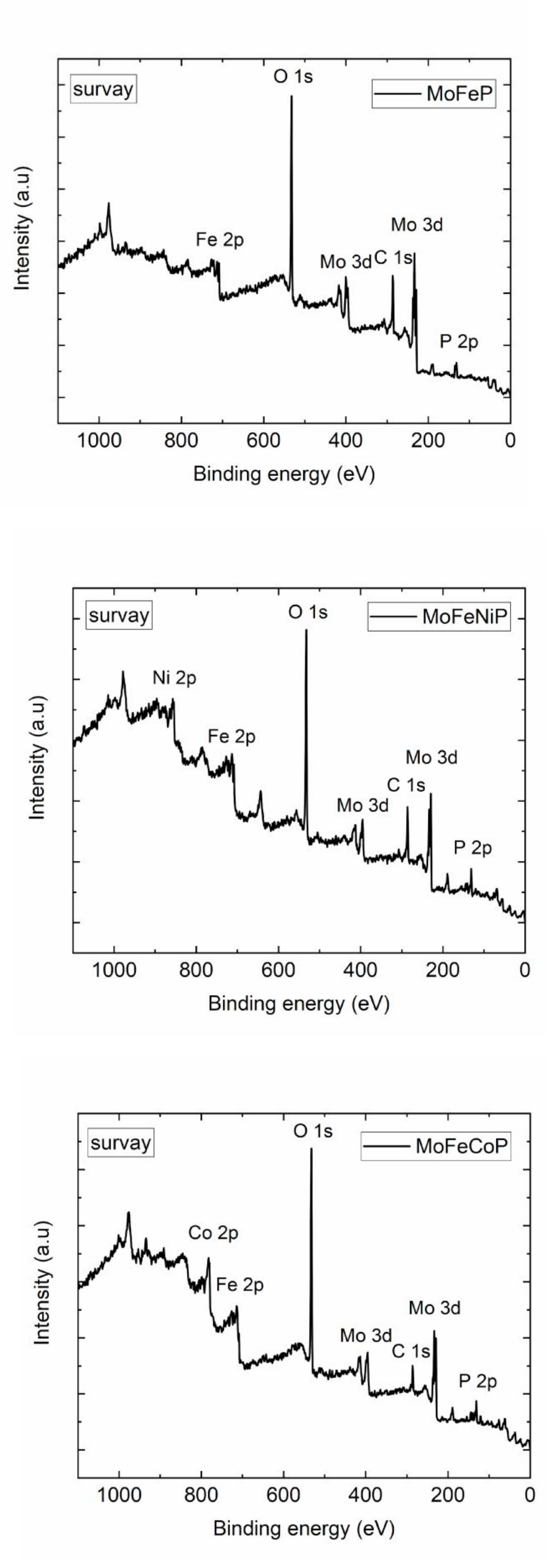

Figure S3 Survey X-ray photoelectron spectra of investigated samples

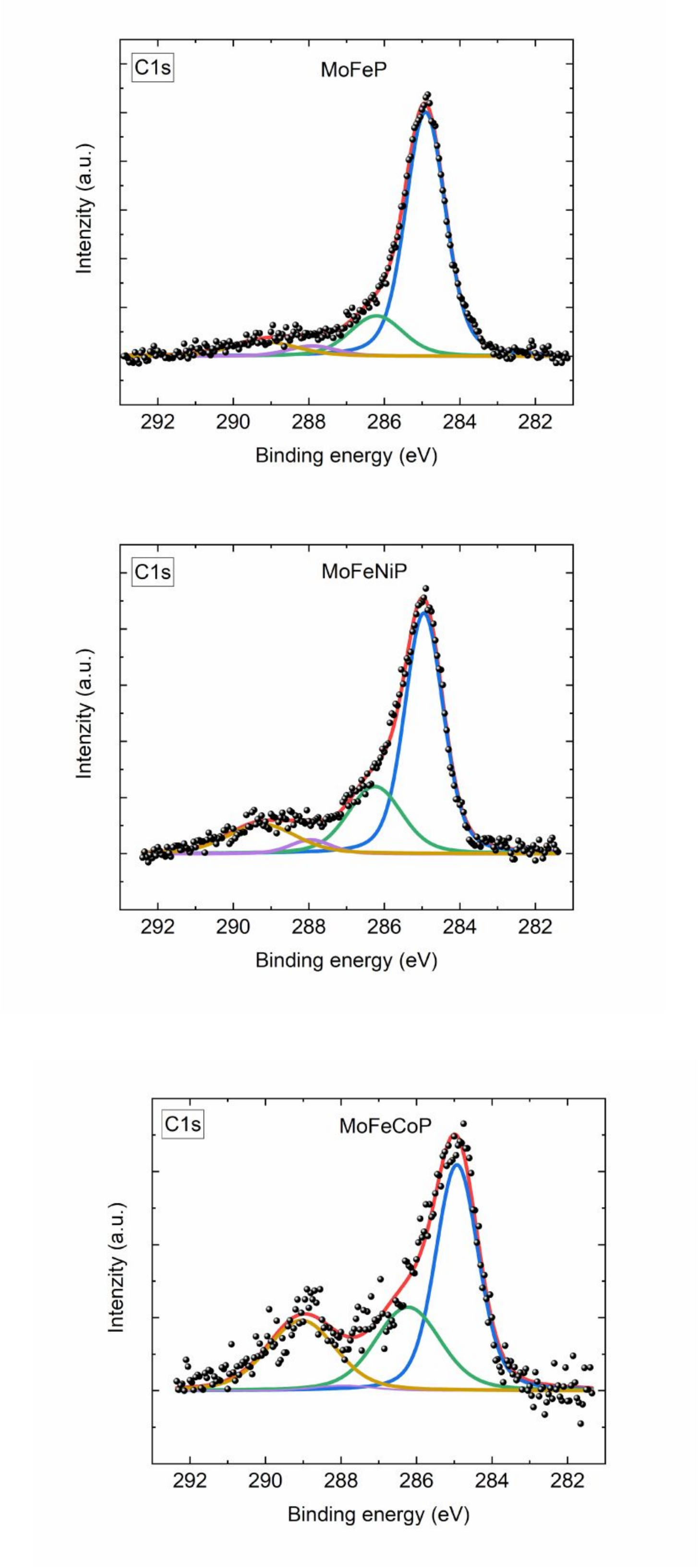

Figure S4 Detailed X-ray photoelectron C1s spectra of investigated samples with deconvolution.

Methods and data for electrochemical characterization

The electrochemical experiments were conducted using a Vionic potentiostat/galvanostat (Metrohm, Switzerland). The potential was calculated with respect to the reversible hydrogen electrode (RHE) according to Eq.(1).

$$E_{vs\,RHE} = E_{vs\,Ag/AgCl} + E_{Ag/AgCl} + 0.0591 \times pH \quad \text{(S1)}$$

Table S1 Values of overpotentials at current densities of -10, -20, and -50 mA.cm^{-2} (η_{-10}, η_{-20}, η_{-50}) and Tafel slopes (b) in 1 M KOH for all samples

Sample	η_{-10} [mV]	η_{-20} [mV]	η_{-50} [mV]	b [mV dec^{-1}]
Pt RDE	-100	-128	-178	51
MoFeCoP	-258	-292	-357	82
MoFeP	-337	-372	-440	92
MoFeNiP	-421	-467	-554	89
GCE	-718	-756	-851	112

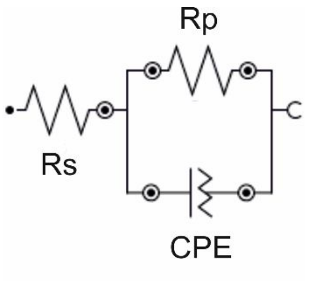

Figure S5 The circuits used for fitting EIS measurements in 1M KOH

The double-layer capacitance of real electrode-electrolyte interfaces is often well described by a Constant Phase Element (CPE) instead of a simple capacitor. CPE has impedance Z_{CPE} defined by the Eq. 2:

$$Z_{CPE} = (1/Y_0)/(j\omega) \quad \text{(S2)}$$

where Y_0 and α (α ≤ 1) are CPE constants and ω is angular velocity. When α = 1, then CPE behaves like a capacitor with capacitance C, i.e. $Y^0 = C$.

χ^2 indicates an error in EIS fit

The double layer capacitance (Cdl) values were determined using the cyclic voltammetry (CV) method at scan rates of 20, 50, 100, 200, and 400 mV/s. The electrochemically active surface area (ECSA) of the samples is typically estimated using a simple CV approach. The ECSA of a catalyst sample is calculated from the the double layer capacitance (Cdl) according to Eq S3:

$$ECSA = \frac{Cdl}{Cs} \qquad \text{(S3)}$$

However, determining the exact surface area of the material is challenging due to the unknown capacitive behavior (Cs) of the specific catalysts. Nevertheless, relative surface areas can be reliably estimated, as the Cdl is expected to be linearly proportional to the effective active surface area. The Cdl is determined by plotting the charging current density around the open circuit potential (Δj), calculated according to Eq. S4 against the scan rate.

$$\Delta j = (ja - jc)/2 \qquad \text{(S4)}$$

ja and jc are oxidation and reduction current density at open circuit potential, respectively.

Effective differential capacitance C_{eff} of a circuit consisting of parallelly connected resistor (with resistance R_{p}) and CPE (with impedance Z_{CPE}) can be calculated using the formula:

$$C_{eff} = Y_0^{(\frac{1}{\alpha})} R_p^{(\frac{1}{\alpha}-1)} \qquad \text{(S5)}$$

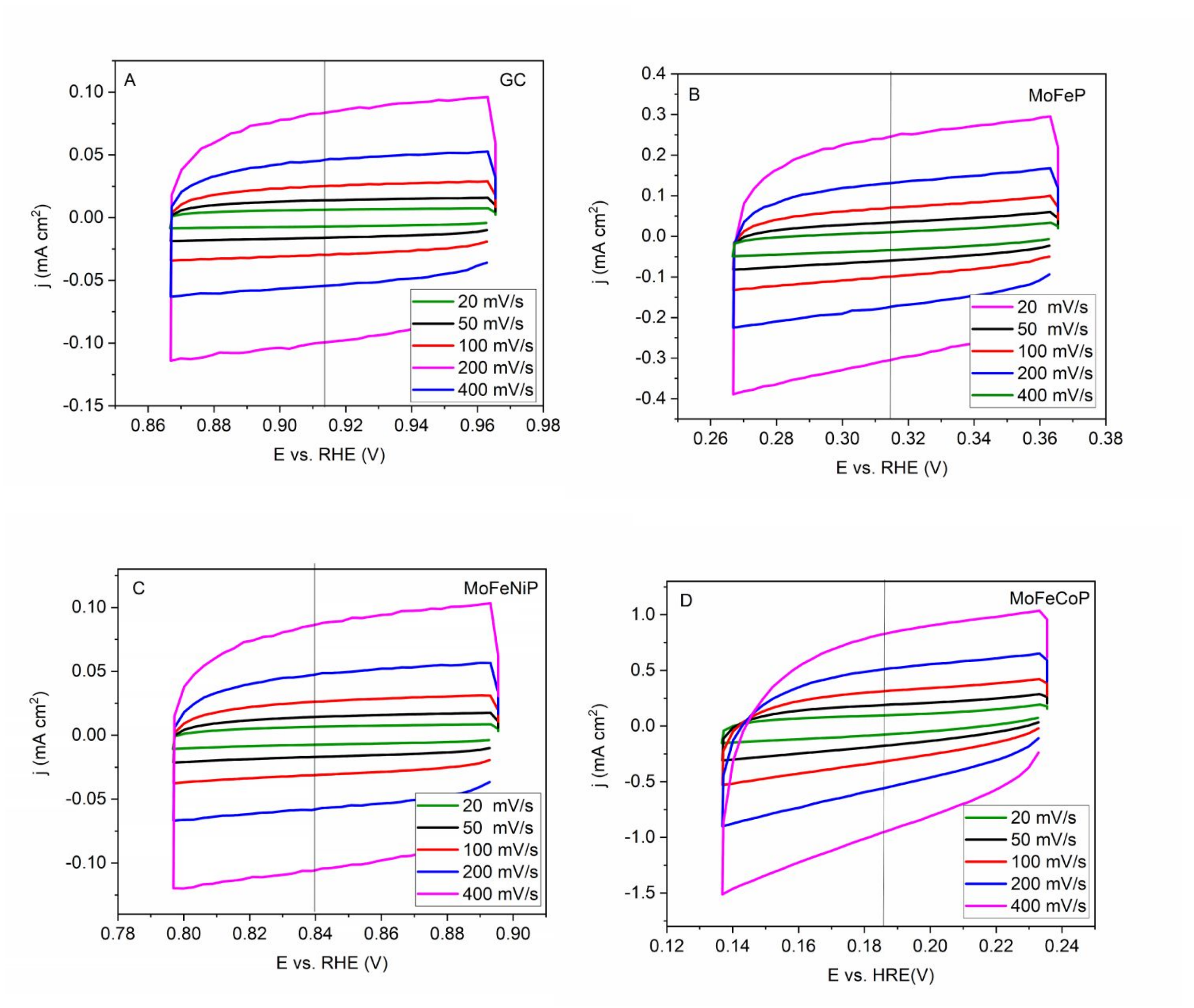

Figure S6 CV for ECSA measured for A) GC, B) MoFeP, C) MoFeNiP, D) MoFeCoP in 1M KOH

Recalculation of the potentials measured with respect to Ag/AgCl reference electrode (at temperature *T*), i.e. $E^T{}_{vs\ Ag/AgCl}$ to RHE scale (at temperature *T*), i.e. $E^T{}_{vs\ RHE}$:

$$E^T{}_{vs\ RHE} = E^T{}_{vs\ Ag/AgCl} + E^T{}_{Ag/AgCl} + \frac{RT}{F} ln\ (10)\ pH \qquad \text{(S6)}$$

$E^T{}_{\text{Ag/AgCl}}$ values can be found in Table S2

Table S2. Standard potential of reference E^T $_{Ag/AgCl,\ 3\ M\ KCL}$ electrode vs RHE with increasing temperature [1].

T [K]	$E_{Ag/AgCl}$ [V]
298.15	0.207
303.15	0.2034
308.15	0.1998
313.15	0.1961
318.15	0.1923
323.15	0.1884
328.15	0.1844
333.15	0.1803

[1] https://www.meinsberger-elektroden.de/en/ueberblick/potential.html

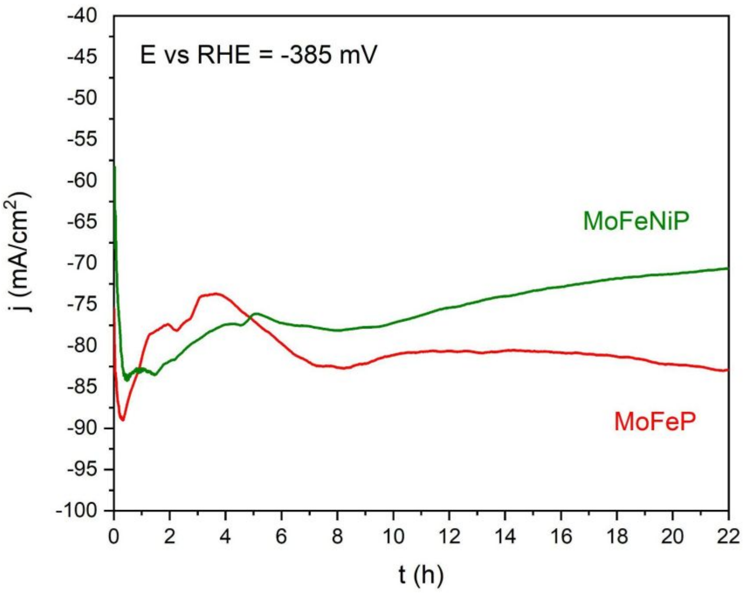

Figure S7 Chronoamperometric stability test (I-t) of MoFeP and MoFeNiP at –385 mV vs. RHE for 22 h at 500 RPM.

The long-term stability of the MoFeP and MoFeNiP catalysts was evaluated under constant potential at -385 mV vs RHE for 22 h. As shown in **Figure S7**, both catalysts exhibit an initial drop in current density followed by stabilization. MoFeNiP maintains a higher and more stable current density throughout the test compared to MoFeP, indicating enhanced

durability upon incorporation of Ni. The gradual increase in current density observed for MoFeNiP suggests possible activation behavior, while MoFeP shows a slight deactivation trend over time.

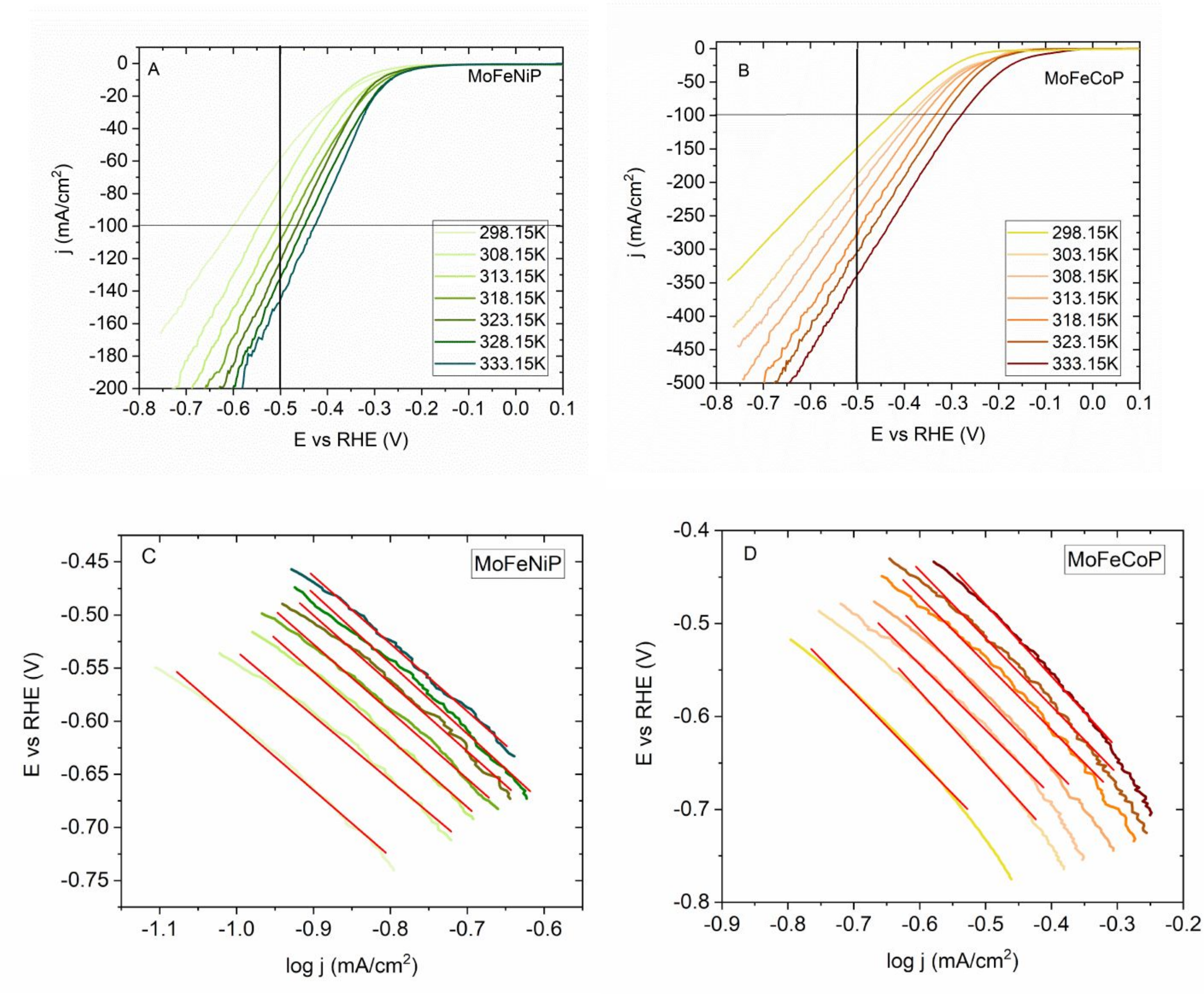

Figure S8 LSV curves for (a) MoFeNiP and (b) MoFeCoP at different temperatures in 1 M KOH. Tafel plots for (c) MoFeNiP and (d) MoFeCoP derived from the LSV curves at various temperatures, scan rate 10 mV s^{-1}.

Table S3. Comparison of the HER performance of multimetallic TMPs with recent literature reports, η_{-10HER} – electrode overpotential at -10 mA cm^{-2}, b_{HER} – Tafel slope

Catalyst	Medium	η_{-10HER} (mV)	b_{HER} (mV dec^{-1})	ref
MoFeP	0.5M H_2SO_4	-132	81	1
MoFeP	1M KOH	-142	73	1
NiFeMoP/CW ♣	1M KOH	-70	52	2
FeS/MoP	0.5M H_2SO_4	-68	66	3
FeS/MoP	1M KOH	-51	105	3
CoP-FeP-MoP_4/NC♥	1M KOH	-75	98	4
$Fe_{0.25}$ - CoP	1M KOH	-111	62	5
Mo,Fe - Ni_3P	1M KOH	-22, -103 $^{\eta100}$	80	6
FeNiMoP/NF ♠	1M KOH	-98	73	7
Mo-FeNiP NTs/NF ♦	1M KOH	-30, -151$^{\eta100}$	76	8
FeNiP@NC •	0.5M H_2SO_4	~-275	149	9
CoNiP@NC	0.5M H_2SO_4	-220	80	9
FeCoP@NC	0.5M H_2SO_4	-108	68	9
FeCoNiP@NC	0.5M H_2SO_4	-93	51	9
FeNiP@NC	1M KOH	-214	111	9
CoNiP@NC	1M KOH	-204	75	9
FeCoP@NC	1M KOH	-289	64	9
FeCoNiP@NC	1M KOH	-187	52	9
MoFeCoP	1M KOH	-285	83	This work

$^{\eta100}$ – HER overpotential (mV) at -100 mA cm^{-2}, ♣ NiFeMo-P/CW (CW- carbonized wood), ♥ CoP-FeP-MoP_4/NC (Triphasic heterostructure on N-doped carbon nanofibers), ♠ FeNiMoP/NF (FeNiMoP grown on nickel foam NF), ♦ Mo-FeNiP NTs/NF (Mo-FeNiP nanotubes on Ni foam), • FeNiP@NC (N/P-codoped Fe/Co/Ni-containing graphene-based material by deriving metal–organic frameworks (MOFs)

References

(1) Guboova, A.; Orinakova, R.; Streckova, M.; Podrojkova, N.; Parackova, M.; Milkovic, O.; Medvecky, L.; Girman, V.; Bystron, T. Bimetallic MoFe Phosphide Catalysts for the Hydrogen Evolution Reaction. *Electrochim. Acta* **2024**, *506* (July), 145008. https://doi.org/10.1016/j.electacta.2024.145008.

(2) Xu, Q.; Hua, J.; Wang, Y.; Cheng, G. Beechwood-Derived NiFeMo-P/CW Hierarchically Porous Bifunctional Electrocatalysts for Efficient Water Splitting. *J. Power Sources* **2025**, *635*, 236471. https://doi.org/10.1016/j.jpowsour.2025.236471.

(3) El-Refaei, S. M.; Russo, P. A.; Schultz, T.; Koch, N.; Pinna, N. Dual Doping of MoP with M(Mn,Fe) and S to Achieve High Hydrogen Evolution Reaction Activity in Both Acidic and Alkaline Media. *ChemCatChem* **2021**, *13* (20), 4392–4402. https://doi.org/10.1002/cctc.202100856.

(4) Cao, M.; Li, B.; Cao, Y.; Li, Y.; Tian, R.; Shen, Q.; Xie, W.; Gu, W. Co–Fe–Mo Phosphides' Triphasic Heterostructure Loaded on Nitrogen-Doped Carbon Nanofibers by Electrospinning as Efficient Bifunctional Electrocatalysts for Overall Water Splitting. *ACS Appl. Mater. Interfaces* **2025**, *17* (10), 15259–15273. https://doi.org/10.1021/acsami.4c17441.

(5) Yang, Q.; Dai, H.; Liao, W.; Tong, X.; Fu, Y.; Qian, M.; Chen, T. Construction of Fe-Doped CoP with Hybrid Nanostructures as a Bifunctional Catalyst for Overall Water Splitting. *Dalt. Trans.* **2021**, *50* (48), 18069–18076. https://doi.org/10.1039/D1DT03292E.

(6) Pawar, S. M.; Aqueel Ahmed, A. T.; Lee, C. H.; Babar, P. T.; Kim, J. H.; Lee, S. U.; Kim, H.; Im, H. Experimental and Theoretical Insights into Transition-Metal (Mo, Fe) Codoping in a Bifunctional Nickel Phosphide Microsphere Catalyst for Enhanced Overall Water Splitting. *ACS Appl. Energy Mater.* **2021**, *4* (12), 14169–14179. https://doi.org/10.1021/acsaem.1c02930.

(7) Wei, Y.; Shin, C.-H.; Gyan-Barimah, C.; Tetteh, E. B.; Park, G.; Yu, J.-S. Positive Self-Reconstruction in an FeNiMo Phosphide Electrocatalyst for Enhanced Overall Water Splitting. *Sustain. Energy Fuels* **2021**, *5* (22), 5789–5797. https://doi.org/10.1039/D1SE01541A.

(8) Wang, X.; Zhou, J.; Cui, W.; Gao, F.; Gao, Y.; Qi, F.; Liu, Y.; Yang, X.; Wang, K.; Li,

Z.; Yang, Y.; Chen, J.; Sun, W.; Sun, L.; Pan, H. Electron Manipulation and Surface Reconstruction of Bimetallic Iron–Nickel Phosphide Nanotubes for Enhanced Alkaline Water Electrolysis. *Adv. Sci.* **2024**, *11* (26), 1–11. https://doi.org/10.1002/advs.202401207.

(9) Sun, J.; Li, S.; Zhang, Q.; Guan, J. Iron–Cobalt–Nickel Trimetal Phosphides as High-Performance Electrocatalysts for Overall Water Splitting. *Sustain. Energy Fuels* **2020**, *4* (9), 4531–4537. https://doi.org/10.1039/D0SE00694G.
